# The Book World of Medicine and Science

**Published:** 1907-03-02

**Authors:** 


### The Book World of Medicine and Science.

Pediatrics: the Hygiene and Medical Treatment of Children. By Thos. Morgan Rotch, M.D. Fifth Edition. (Philadelphia and London: J. B. Lippincott Company. 1906. Pp. xxv and 1,060. Price 25s. net.)

Professor Rotch's manual has received wide recognition as one of the most complete of the numerous works dealing systematically with the study of disease in children. In the new edition the reputation of the book is well maintained, and its sphere of usefulness may be expected to be enlarged. As might have been anticipated from the investigations of the author, the subject of infant feeding receives particular attention, and full particulars are supplied of the elaborate care which is necessary to secure a pure milk supply. Detailed information is also given of the system of percentage feeding which Professor Rotch has done so much to extend in America, and to which more attention might with advantage be paid in this country. The illustrations are numerous and effective. As a systematic work of reference the book may be consulted with confidence.

Tumours, Innocent and Malignant; Their Clinical Characters and Appropriate Treatment.—By J. Bland Sutton, F.R.C.S. Fourth edition. (Messrs. Cassell and Co., Ltd. 8vo. Lond. 1906, pp. xii. and 675, with 355 engravings. Price 21s.)

We welcome a new and revised edition of Mr. Bland Sutton's book on Tumours. It is written so crisply as to make its perusal a pleasure, whilst at the same time it covers so much ground, gives so many references, and contains so much accurate information, as to leave the English-speaking medical practitioner without any excuse for ignorance upon the topics with which it deals. Pathology in the more restricted sense of morbid anatomy has always had worthy exponents in English. Hunter, indeed, had no charm of manner, but Baillie, and after him Paget, gave a grace to the subject by their writings, and this has been admirably maintained by Mr. Bland Sutton. Surgical pathology at the present time is suffering a partial eclipse, owing to the predominance of bacteriological teaching in the medical schools. There can be no doubt, however, that a man who works through, and knows, the specimens in a large museum will give better diagnoses than one who has spent an equal amount of time in a bacteriological laboratory. The claims of the museum were formerly paramount; they are now relegated to a position of secondary importance. Mr. Bland Sutton's book is essentially a museum book. Into it he has skilfully worked the latest investigation on the histology of tumours with the general results of the organised laboratory research for the causative agent of cancer. Interspersed, too, throughout the volume, and in a true Hunterian spirit, are many illustrative observations taken from comparative anatomy and ethnology, for it is only by the widest knowledge that morbid actions can be explained. On all points Mr. Bland Sutton's teaching is sound and thorough, both as to theory and practice. He has no fads, and what he thinks he writes in downright English. He adds to each chapter a short bibliography of papers which can be easily consulted by any reader who has access to a moderate-sized medical library, and by these references anyone who is interested can follow up a particular subject to its source. The present edition contains, in addition to the chapter on the cancer question—to which, by the way, Mr. Bland Sutton might have added some remarks upon the influence of locality on recurrence—some valuable observations on tumours of the ovary and testicle, and a chapter on heterotopic teeth. This chapter well repays perusal. It deals with ovarian teeth, with the mastoid or tympanic teeth of horses, and with the cervical teeth of ruminants.

The book is excellently illustrated with blocks made for the most part by Messrs. Butterworth from the drawings of Messrs. Berjeau, Balcombe, Lewin, and other artists. More than fifty additional illustrations occur in the present edition. There is a table of contents, an index of tumours, and an index of organs.

International Clinics. Vols. II. and III. Sixteenth Series. (Philadelphia and London: J. B. Lippincott Company. 1906.)

The issue of these quarterly volumes of illustrated clinical lectures and original articles dealing with the various departments of medical and surgical practice, has now been continued for a considerable number of years, and the editors are to be congratulated on the high standard of merit the publication maintains. With but few exceptions the lectures are all of interest and value and may be read with profit by those engaged in actual practice. Particularly do they appeal to general practitioners, as they include all varieties of professional work and deal for the most part with questions of diagnosis and treatment. A well-planned and clearly-stated clinical lecture has many advantages which a systematic text-book cannot claim. It deals with an individual position and difficulty rather than with general rules and statements, and thus has a note of actuality which is often absent from the less detailed discussions of the text-book. By such lectures, post-graduation study, whatever may be true of the education of students, is most effectively conducted, and we therefore commend the present volumes as well worthy of the attention of all practitioners anxious to increase the efficiency of their work.

